# Research on Interlayer Toughening and Damage Detection of Laser-Induced Graphene and Short Kevlar Fibers Aramid Fiber/Epoxy Resin Composites

**DOI:** 10.3390/polym16233380

**Published:** 2024-11-30

**Authors:** Baolai Wang, Weidong Tian, Chao Wang, Qi Wang

**Affiliations:** Yantai Research Institute, Harbin Engineering University, Yantai 264000, China; wangbaolai1979@sina.com (B.W.); 18066241035@163.com (C.W.); 13314486617@163.com (Q.W.)

**Keywords:** laser-induced graphene, short Kevlar fibers, aramid fiber/epoxy resin composites, fracture toughness, tensile strength, damage detection

## Abstract

The poor interlaminar fracture toughness is a critical limiting factor for the structural applications of aramid fiber/epoxy resin composites. This study investigates the effects of laser-induced graphene (LIG) and short Kevlar fibers on the interfacial toughness and damage detection of aramid composite materials. Mode II tests and tensile tests were conducted to evaluate mechanical properties and damage detection using the piezoresistive characteristics of LIG. The results indicate that LIG combined with short Kevlar fibers significantly enhances the interfacial toughness of the composites, achieving a 381.60% increase in initial Mode II fracture toughness. Although LIG reduced the tensile strength by 14.02%, the addition of short Kevlar fibers mitigated this effect, preserving the overall mechanical performance. Scanning electron microscopy (SEM) analysis revealed enhanced toughening mechanisms, including increased surface roughness, altered crack propagation paths, and fiber bridging. Additionally, LIG enabled real-time damage monitoring, showing a significant increase in resistance upon delamination or crack propagation and a marked increase in resistance upon the tensile fracture. This research indicates that the synergistic effects of LIG and short Kevlar fibers not only enhance the interlaminar toughness of aramid composites but also provide a novel strategy for effective damage detection in fiber-reinforced materials.

## 1. Introduction

Aramid fibers possess excellent flame resistance, heat resistance, high toughness, impact resistance, and fatigue resistance. High-performance composites made from aramid fibers are widely used in various fields, including sporting goods, aerospace, marine applications, and ballistic protection, particularly in applications such as helmets and armored vehicles [1,2]. However, the smooth and inert surface of aramid fibers results in poor adhesion with the resin matrix, which adversely affects the interlaminar mechanical properties and the overall load-bearing capacity of the structure, thereby limiting the practical application of aramid fibers [3,4,5,6]. Therefore, it is crucial to conduct in situ damage detection and interlaminar toughening studies for aramid fiber-reinforced composites.

Over the past 30 years, extensive research has been conducted on interlaminar toughening by scholars. Sela et al. [7] enhanced the fracture toughness of structures by doping second phases such as ceramics and rubber to toughen the resin. Subsequently, resin toughening techniques have continuously developed, with thermoplastic resins, carbon nanotubes, and thermoplastic nanofibers utilized as reinforcing materials for toughening modifications [8,9,10]. However, these methods can adversely affect the mechanical properties of the resin matrix. Additionally, Z-fiber toughening techniques such as Z-pin, 3D weaving, and stitching technology have been proposed and applied to delay delamination in composites [11,12,13]. Nonetheless, these approaches face challenges such as high processing difficulty, expensive manufacturing costs, and potential impacts on the in-plane mechanical properties of the structure. In response to the numerous issues associated with these methods, many researchers have shifted their focus to interlaminar toughening, which includes particle toughening, film toughening, and short fiber toughening. Minh Hoang Nguyen [14] introduced toughening particles into the interlayer of carbon fiber composites, resulting in an approximately 100% increase in the ultimate shear strength. Salvatore Giacomo Marino et al. [15] inserted 30% and 60% thermoplastic acrylonitrile butadiene styrene (ABS) overlapping films between the layers of carbon/glass–epoxy composites. The pseudoplastic strain increased to 0.67% and 0.74%, respectively, while the carbon fiber volume fraction decreased by 12.7% and 16.5%, demonstrating improved interlaminar Mode II fracture toughness. Both methods can improve interfacial toughness; however, they present significant processing challenges.

Compared to other toughening methods, short fiber toughening only requires spreading short fibers between the panels, which has garnered significant attention from researchers due to its operational simplicity. Scholars have investigated the effects of inserting various types and contents of short fibers on interlaminar toughness and in-plane mechanical properties. Dong Quan et al. [16] found that incorporating ultraviolet-irradiated poly-etherether-ketone (PEEK) fibers into the interlayers of carbon fiber composites significantly improved Mode I fracture toughness from 185 J/m^2^ to 726 J/m^2^. Melike Kilicoglu et al. [17] demonstrated that inserting polyamide 6/polycaprolactone (PA6/PCL) mixed fibers between the layers of carbon fiber composites could markedly enhance Mode I fracture toughness, particularly achieving optimal results at a 60/40 ratio, where the initiation and propagation toughness increased by 69% and 59%, respectively. Park et al. [18] indicated that surface-modified short Kevlar fibers significantly increased the Mode II fracture toughness of carbon fiber composites with increasing crack length, from around 1.0 kJ/m^2^ to 3.0–4.0 kJ/m^2^. Zheng Hao et al. [19] concluded that short Kevlar fibers exhibited the best toughening effect, with the highest Mode I fracture toughness reaching 1.336 kJ/m^2^, an increase of 94.8%, determining that the fiber length was 6 mm, while finding that short fiber insertion did not affect in-plane mechanical properties. A comprehensive analysis reveals that current short fiber toughening technology is primarily applied to carbon fiber-reinforced epoxy composites, with short Kevlar fibers demonstrating the most effective toughening performance. In contrast, aramid fiber composites face limitations due to inadequate surface activation, which hinders effective bonding with the resin matrix. Therefore, it is crucial to modify the surface of aramid fibers to enhance roughness and improve their bonding efficacy.

Currently, the modification methods for aramid fibers primarily include physical and chemical modifications. These methods involve the application of plasma or the introduction of specific chemical agents to improve interfacial properties. However, these approaches may damage aramid fibers and reduce their tensile strength [20,21]. In 2014, LIN [22] first employed a carbon dioxide infrared laser system to directly generate laser-induced graphene (LIG) on the surface of commercial polyimide films (PI). This method has garnered significant attention due to its simplicity and the fact that it does not require chemical modification. Jalal Nasser et al. [23] demonstrated that transferring LIG to the surface of carbon fibers and found that the LIG layer generated at 200 DPI pulse density increased Mode I and Mode II fracture toughness by 41% and 69%, respectively, while maintaining tensile performance, confirming the effectiveness of LIG in improving interface toughness. LIG also exhibits excellent piezoresistive properties and high electrical conductivity, enabling resistance-based sensing methods for the detection of strain or damage in insulating materials. Typically, an electric current is applied to measure voltage, allowing resistance changes to be calculated based on Ohm’s law; a sudden increase in resistance is detected when the material sustains damage. LA Groo et al. [24] integrated LIG into glass fibers to monitor damage accumulation and propagation during tensile fatigue loading through resistance changes. This resistance-based sensing differs from using external sensors or embedded fiber sensors as it does not add to the structural volume or weight [25,26]. Subsequently, Chyan et al. [27] discovered that LIG could be directly generated on the surface of aramid fibers, facilitating its application in aramid fiber composites. Lori Anne Groo et al. [28] generated graphite structures directly on the surface of aramid fibers, demonstrating that these structures could enable in situ monitoring of tensile and bending strains by measuring resistance. Given the critical ballistic performance of aramid fibers, Kelsey Steinke et al. [29] utilized LIG for embedded impact sensing in aramid fiber-reinforced composites, employing resistance measurements to obtain information about the impact severity. Since the quality of LIG is influenced by factors such as laser power, scanning speed, and pulse frequency, Iman Naseri et al. [30] investigated the effects of these laser parameters on the quality of LIG on the surface of aramid fibers, indicating a significant impact of laser settings on LIG quality. However, while LIG has been shown to enhance interlaminar fracture toughness and serve as a resistance sensor, the effects of short Kevlar fibers combined with LIG on aramid fiber-reinforced composites remain unexplored.

This study investigates the effects of LIG and short Kevlar fibers on the mechanical properties of aramid fiber/epoxy resin composites while employing LIG for damage tracking during loading tests. Graphene was generated on the surface of aramid fibers using laser induction, and short Kevlar fibers were introduced into the interlayers. The end-notched flexure (ENF) test was utilized to evaluate the impact of these modifications on the interfacial fracture toughness of the composites, determining the capability of LIG to track delamination damage. Scanning electron microscopy (SEM) was employed to observe the fracture surfaces, revealing the toughening mechanisms involved. Additionally, tensile tests were conducted to analyze the influence of short Kevlar fibers and LIG on the in-plane mechanical properties of the composites while also assessing the ability of LIG to monitor damage induced by tensile loading.

## 2. Materials and Methods

### 2.1. Materials

In this study, a 1500D meta-aramid woven aramid fiber fabric produced by DuPont was selected as the panel material, with an areal density of 200 g/m^2^. Due to its high strength and abrasion resistance, this material is widely used in various applications. The smooth surface of the aramid fibers means that the adhesive strength at the fiber-matrix interface primarily depends on the roughness of the fiber surface after treatment and the bridging effect of the inserted short fibers.

The epoxy resin system used in this study was prepared by mixing Z105 epoxy resin (from West System) with slow hardener 206 (from West System) in a ratio of 5:1, ensuring thorough impregnation of the aramid fiber fabric surface. To ensure adequate bonding between the short Kevlar fibers and the aramid fiber panel, it is imperative to use sufficient epoxy resin to guarantee that the short fibers are completely wetted within the voids between the panel fibers.

### 2.2. Short Aramid Fibers Preparation

**Figure 1:**
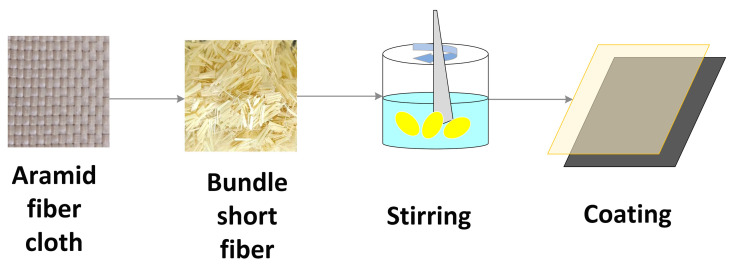
Preparation process of short Kevlar fiber film.

The short fibers utilized in this study are Kevlar 49, produced by DuPont. According to the research conducted by Zheng Hao et al. [19], the maximum fiber bridging region and the best toughening effect occur when the length of the short fibers is 6 mm. Therefore, Kevlar 49 fibers were cut to a length of 6 mm, with a length tolerance maintained within ±0.5 mm. The chopped short Kevlar fiber bundles were then placed in a blender equipped with dull blades and mixed at high speed until the fibers transitioned from bundles to a uniformly dispersed fluff-like consistency. Finally, the short Kevlar fibers were thoroughly impregnated with the mixed epoxy resin to form a uniformly distributed short fiber film, based on the desired fiber density. For instance, in this study, short Kevlar fibers treated with epoxy resin were laid between the aramid fiber panels, resulting in a short Kevlar fiber film with a mass per unit area of 6 g/m^2^ after curing. The preparation process of the short Kevlar fiber film is illustrated in Figure 1.

### 2.3. Generation of LIG on Aramid Surface and Characterization

**Figure 2:**
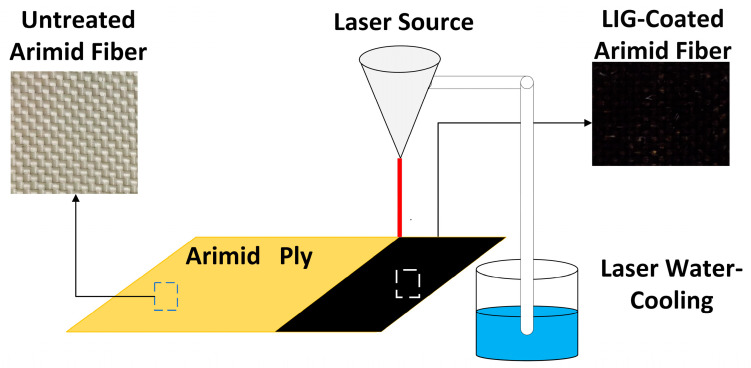
Generation of graphene on the surface of aramid fibers.

According to the research by Kelsey Steinke et al. [29], graphene was generated on the surface of aramid fibers. Prior to laser treatment, aramid fabric sheets were cut to dimensions of 300 mm × 300 mm, with a cutting tolerance maintained within 1 mm. The edges were then hardened and fixed to prevent the shedding of fine fibers. The cut aramid fabric was first cleaned in a 95% ethanol solution using electromagnetic waves for 15 min, followed by drying in an oven at 90 °C for 60 min to remove surface impurities and residual moisture. A CO_2_ infrared laser engraving machine (80W-4060, from Ketai Laser, Liaocheng, China) was employed to generate graphene on the surface of the aramid fibers. The laser processing was conducted in raster mode, with a laser power set to 12 W and a scanning speed of 300 mm/s. The distance between the laser output and the aramid fiber surface was maintained at 14 mm. To ensure stable laser output, the laser water-cooling was employed for water circulation temperature control, maintaining the operating temperature of the laser generator within the range of 20–23 °C. This process facilitated the uniform generation of graphene arrays on the surface of the aramid fibers. The preparation process of graphene on the aramid fiber surface is illustrated in Figure 2. After the laser treatment, a Renishaw 2000 high-precision Raman spectrometer, operating at a wavelength of 532 nm, was employed to analyze the surface of the aramid fibers before and after treatment.

### 2.4. Model II Specimen Fabrication

The Mode II fracture toughness of the LIG-coated and short Kevlar fibers that toughen aramid fiber-reinforced composites were assessed following the ASTM D7905. A total of 24 LIG-coated aramid fiber prepregs were fully impregnated with a resin mixture containing short Kevlar fibers. After impregnation, 12 prepregs were arranged with the LIG-coated facing upwards, while the remaining 12 prepregs were oriented with the LIG coating facing downwards, achieving a symmetrical layout. To introduce a pre-crack within the laminate, a 70 mm long Teflon film was inserted between the 12th and 13th layers, resulting in an initial delamination length of 50 mm. After impregnation, the aramid fiber fabric is placed into a 300 mm × 300 mm mold and cured under a constant pressure of 0.3 MPa. The mold is heated from room temperature to 60 °C in 20 min, held at this temperature for 4 h, and then allowed to cool to room temperature naturally. After the epoxy resin fully cured, the 6 mm thick laminate was cut into 190 mm in length and 25 mm in width. A thin layer of white paint was sprayed on the edges of the specimens to facilitate visual observation of the crack initiation and propagation. For in situ resistance measurements, silver paint rings were affixed to each side of the laminate and connected to 33-gauge copper wires with silver paint and electrical tape. This methodology establishes a robust experimental basis for examining the toughening mechanisms in composite materials.

### 2.5. Mode II Testing

The completed specimens were tested using an Instron universal testing machine under quasi-static loading at a rate of 5 mm/min. A three-point bending setup with a 5 kN load cell recorded data, with a span of 70 mm and a crack tip distance of 50 mm from the left support. Loading was ceased once the crack propagated to the load center. For the assessment of failure behavior, the critical strain energy release rate G_C_ was predominantly used to characterize the interlaminar toughness of the composite materials, while the Mode II delamination fracture toughness was calculated using the following equation [31]:

(1) $$G_{\mathrm{II}C}=\frac{9P\delta a^{2}}{2W(2L^{3}+3a^{3})}\times 10^{3}$$

where *P* is the critical load; *δ* is the corresponding critical displacement; *a* is the initial effective crack length; *W* is the specimen width; and 2*L* is the span length.

**Figure 3:**
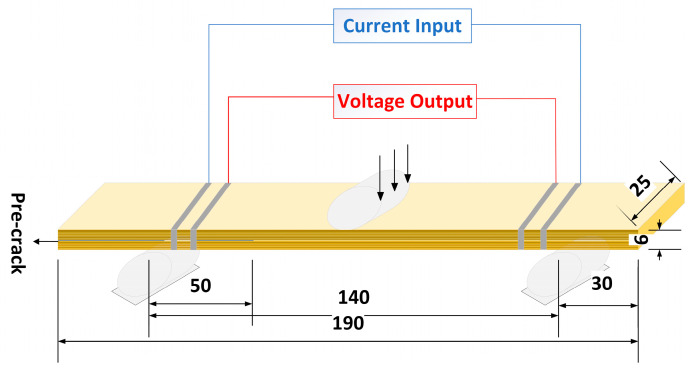
Schematic diagram of Mode II specimens.

During the delamination testing, a 3 mA DC current was applied via a power supply (from Kuaiqu Electron, Shenzhen, China) to the copper wire connected to the outermost silver paint ring. A digital multimeter measured the voltage at the innermost silver paint ring. A schematic of the loading and in situ monitoring setup is presented in Figure 3. After testing, fracture surfaces were analyzed using a TESCAN CLARA (from Tescan, Brno, The Czech Republic) scanning electron microscope (SEM). A representative fracture surface of the panel was selected for gold coating to improve conductivity. The analysis was conducted with an acceleration voltage of 20 kV, and magnification varied from 20× to 1000×.

### 2.6. Tensile Specimen Fabrication

Three layers of LIG-coated aramid fiber fabric were thoroughly impregnated using a mixed epoxy resin containing short Kevlar fibers oriented with the LIG side up, using the same molding process as the Mode II specimens. After the epoxy resin had fully cured, the 0.9 mm thick laminates were cut into specimens measuring 160 mm in length and 12.25 mm in width according to ASTM D3039. A layer of silver paint was applied to the edges of each specimen, and 33-gauge copper wire was connected to facilitate the measurement of electrical resistance changes during tensile testing.

### 2.7. Tensile Testing

**Figure 4:**
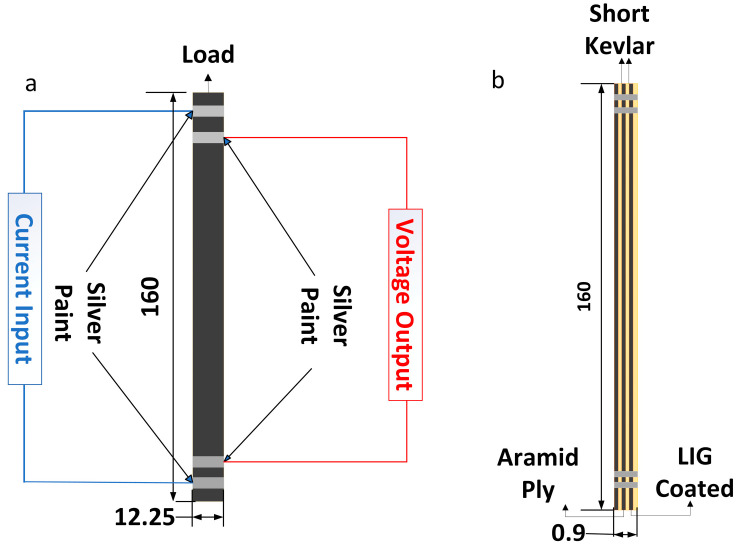
(**a**) Schematic diagram of the tensile specimens (front view); (**b**) Schematic diagram of tensile specimens (side view).

The same loading frame and loading rate used in the Mode II delamination tests were employed until specimen failure occurred. During the tensile testing, resistance measurements were simultaneously taken using the same method as in the delamination tests. A schematic diagram of the loading and in situ monitoring of the tensile specimens is shown in Figure 4.

## 3. Results and Discussion

### 3.1. LIG Chemical Characterization

Raman spectroscopy plays a crucial role in the analysis of graphene due to its ability to accurately characterize the microstructure and chemical composition of the material [32,33]. In particular, Raman spectroscopy effectively identifies key features of graphene, including the D peak, G peak, and 2D peak. The D peak (approximately 1350 cm^−1^) reflects structural defects in graphene, arising from the breathing modes of hybridized carbon atoms. The G peak (approximately 1582 cm^−1^) represents in-plane vibrations of carbon atoms, indicating the strength and integrity of the graphite structure, and is considered the primary characteristic peak of graphene. The intensity ratio between the D peak and the G peak (I_D_/I_G_) serves as an important indicator of the defect density and crystallinity of graphene. The 2D peak (approximately 2700 cm^−1^), also known as the G‘ peak, represents the stacking arrangement of carbon atoms between layers and is independent of structural defects; its full width at half maximum is primarily used to assess the number of graphene layers. Consequently, by analyzing these three characteristic peaks in Raman spectra, we can determine whether graphene has been generated on the surface of aramid fibers and evaluate the quality of the produced graphene.

**Figure 5:**
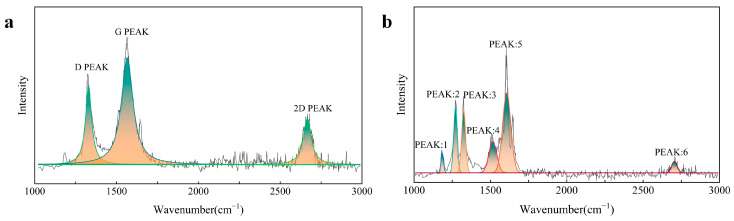
(**a**) Raman spectrum of aramid fibers LIG-coated; (**b**) Raman spectrum of untreated aramid fibers.

Figure 5 presents the Raman spectra of the aramid fiber surface before and after treatment. A comparison reveals that the Raman spectrum prior to treatment (Figure 5b) displays several characteristic peaks of the aramid fibers at 1182 cm^−1^, 1274 cm^−1^, 1327 cm^−1^, 1515 cm^−1^, 1599 cm^−1^, and 1647 cm^−1^ [34]. In contrast, the Raman spectrum after laser induction (Figure 5a) clearly shows the D band, G band, and 2D band of graphene, located at 1325 cm^−1^, 1563 cm^−1^, and 2679 cm^−1^, respectively. Moreover, I_D_/I_G_ indicates that the graphene generated through laser induction exhibits a high degree of graphitization, minimal structural defects, and a high level of crystallinity. Therefore, under these laser parameters, high-quality graphene can be produced on the surface of aramid fibers.

### 3.2. Interlaminar Fracture Toughness

Due to the susceptibility of composite laminates to interlaminar fracture failure, the application of LIG onto the surface of aramid fibers can significantly enhance the Mode II fracture toughness. Furthermore, its piezoresistive properties enable effective monitoring of delamination failure in the laminates.

**Figure 6:**
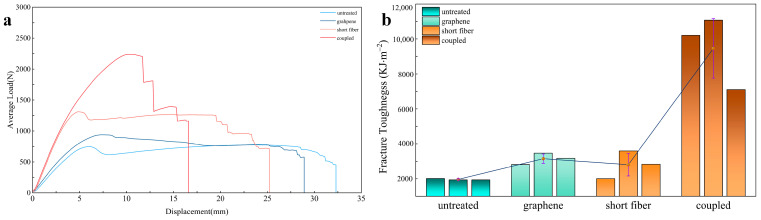
(**a**) Average load displacement curves for the four types of specimens; (**b**) Fracture toughness at the onset of delamination.

This study conducted a detailed analysis of five sets of experimental data, selecting three sets with outstanding results for further evaluation. The average load and average fracture toughness were calculated for these data sets. Figure 6a presents the average load displacement curves for the four types of specimens. It is evident from the figure that all ENF specimens exhibit a similar trend; they demonstrate a linear relationship in the elastic phase before delamination occurs. As the displacement load increases, the curves begin to show nonlinearity, indicating the onset of pre-crack delamination, with the cracks propagating relatively slowly. Once the load reaches its peak, rapid crack propagation occurs, leading to a decrease in load until complete failure of the specimen.

In engineering applications, there is often a greater focus on the toughness value at the delamination initiation state [35]. This study identifies the critical point in the linear phase of the load displacement curve as the initiation point for delamination [36]. This method is straightforward and offers high precision. The average initiation fracture toughness for the four types of specimens was calculated using Equation (1), with results presented in Table 1. The data indicate that the average initiation fracture toughness values for the untreated laminate, laminate only LIG-coated, laminate only short Kevlar fibers toughened, and the hybrid laminate are 1964.46 KJ × m^−2^, 3152.53 KJ × m^−2^, 2809.18 KJ × m^−2^, and 9460.79 KJ × m^−2^, respectively. Correspondingly, the fracture toughness values improved by 60.48%, 43.00%, and 381.60%.

The results indicate that both LIG and short Kevlar fibers can enhance the delamination fracture toughness of laminates. The LIG coating increases the roughness of the aramid fiber surface, thereby improving the adhesion strength between the panel and the resin matrix. It also absorbs energy during interfacial debonding and slip processes, allowing the interfacial layer to better resist crack propagation under shear forces. Moreover, the high modulus of LIG prevents further crack extension through its own elastic deformation, thereby enhancing the fracture toughness of the laminate. In the case of short Kevlar fibers, their inherent toughness allows them to absorb energy through elastic deformation during crack propagation, preventing direct crack penetration, thereby preventing direct crack penetration. Additionally, the fracture and pullout behaviors of the short Kevlar fibers contribute to energy absorption during crack growth, thus enhancing interfacial toughness [35]. However, compared to the laminate containing only LIG, the laminate with only short Kevlar fibers lacks surface modification on the aramid fibers. This smooth fiber surface fails to form effective bonding with the epoxy resin, resulting in a less pronounced bridging effect of the short fibers; thus, the improvement in fracture toughness for the laminate with only short Kevlar fibers is minimal. In contrast, the combined effect of the LIG-coated and short Kevlar fibers significantly enhances the structural fracture toughness.

To better evaluate the synergistic toughening effect of LIG and short Kevlar fibers, the Mode II interlaminar fracture toughness of carbon fiber/epoxy composites containing only LIG generated at a pulse frequency of 200 DPI [23] and those containing only 10 g/m^2^ short Kevlar fibers [37] were compared (Table 2). The results showed that the fracture toughness of the carbon fiber/epoxy composites containing only LIG increased by 69%, while the composites with only short Kevlar fibers exhibited a 61.8% improvement. Both of these values were lower than the 381.79% increase in fracture toughness achieved by the synergistic effect of LIG and short Kevlar fibers. Notably, whether containing only LIG or only short Kevlar fibers, the carbon fiber/epoxy composites exhibited superior toughening effects compared to the aramid fiber/epoxy composites. This can be attributed to the stronger interfacial bonding between carbon fibers and epoxy resin, which facilitates efficient load transfer to the fibers under external forces, thereby enhancing the overall toughness of the composite material.

To further understand the toughening mechanisms of LIG and short Kevlar fibers on the delamination of laminates under Mode II testing, this study employs SEM imaging to observe the fracture surfaces of the specimens.

**Figure 7:**
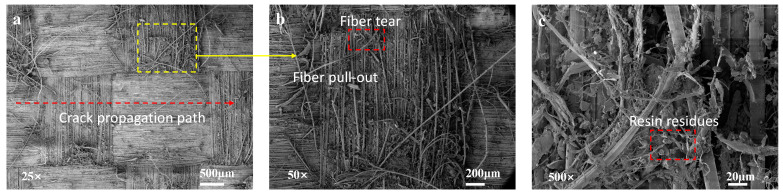
(**a**–**c**) SEM micrographs of the fracture surfaces of untreated specimens.

For the untreated specimens, the fracture surfaces exhibit relatively clean characteristics, with minimal residue of the resin matrix. This phenomenon can be attributed to the extremely smooth surface of the untreated aramid fibers, which hinders the formation of a strong interfacial bond with the resin matrix. Interfacial debonding is identified as the primary cause of failure for the untreated specimens. Additionally, it can be observed that the transverse fibers are arranged in an orderly manner, while the longitudinal fibers show slight signs of damage, accompanied by a small number of fiber breakage, pull-out, and resin residue. This behavior can be explained by the stress distribution characteristics during loading: transverse fibers primarily bear shear stresses, and their orientation is perpendicular to the loading direction, resulting in relatively lower tensile stresses. As the resistance along the transverse fiber direction is minimal, crack propagation tends to occur along this path. In contrast, longitudinal fibers directly face the predominant tensile stresses, making them more susceptible to fracture or damage during loading (Figure 7).

**Figure 8:**
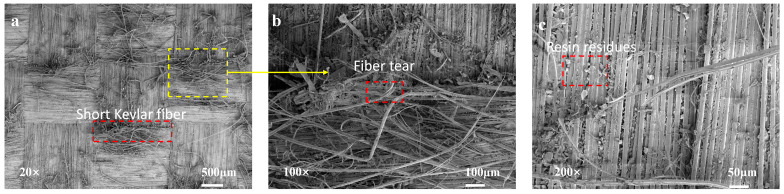
(**a**–**c**) SEM micrographs of the fracture surfaces of only short Kevlar fiber specimens.

For the specimens toughened only by short Kevlar fibers, observation of the fracture surface clearly reveals the presence of short fiber breakage bundles in both longitudinal and transverse directions, while the existing aramid fibers showed no significant damage in either direction. These micro-damage mechanisms partially obstruct further crack propagation, thereby effectively enhancing the fracture toughness of the specimens. However, given that no modification treatment was applied to the aramid fiber surfaces, the interfacial bonding between the short Kevlar fibers, the matrix, and the panels remains weak, as evidenced by the minimal resin residue on the fracture surfaces post-failure. This limitation restricts the full realization of the toughening effect (Figure 8).

**Figure 9:**
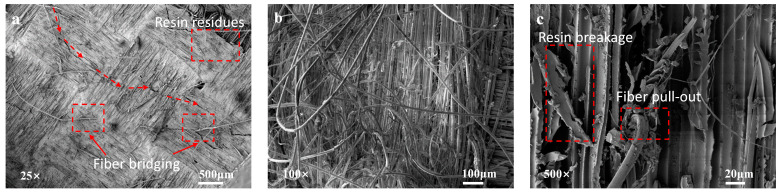
(**a**–**c**) SEM micrographs of the fracture surfaces of only LIG-coated specimens.

In comparison, the fracture surfaces of the specimens only LIG-coated exhibit a significant amount of resin matrix residue, which appears in a plate-like morphology adhered to the surface of the aramid fibers. This surface shows a higher roughness compared to the untreated specimens and those toughened with short Kevlar fibers. This phenomenon can be attributed to the uniform coverage of the LIG coating on the surface of the aramid fibers after treatment, resulting in increased micro-roughness. The enhancement in surface roughness promotes effective wetting between the fibers and the matrix, leading to the formation of a robust interlocking structure. It is noteworthy that the resin matrix on the fracture surfaces displays crack propagation in both the longitudinal and transverse fiber directions, indicating that crack growth is not confined to the longitudinal fiber direction but also involves the transverse direction. This complex crack path increases the length and tortuosity of the fracture trajectory, further dissipating fracture energy and effectively enhancing interlaminar toughness. Additionally, the fiber bridging phenomena observed on the matrix surface reveal the effective connecting capability of the LIG coating between adjacent layers, contributing significantly to the toughening effect (Figure 9).

**Figure 10:**
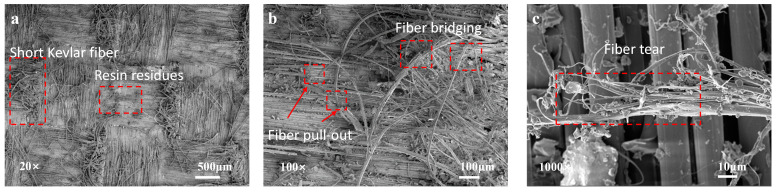
(**a**–**c**) SEM micrographs of the fracture surfaces of combined specimens.

The fracture surfaces of the specimens containing LIG-coated and toughened short Kevlar fibers clearly reveal the presence of epoxy resin and bundles of short fibers. This phenomenon can be attributed to the uniform distribution of the LIG coating on the surface of the aramid fibers, which promotes effective wetting between the aramid fibers and the resin matrix, consequently significantly enhancing the interfacial bonding strength. Furthermore, the incorporation of short Kevlar fibers leads to frequent fiber pull-out and bridging phenomena, resulting in a more complex crack propagation path. Notably, the short Kevlar fibers exhibit characteristics of longitudinal fracture failure, indicating that the Kevlar fibers, due to their exceptional toughness, effectively absorb a substantial amount of energy through elastic deformation, thereby further enhancing the toughening performance of the material. Additionally, the fracture sites of the short Kevlar fibers clearly show residual resin matrix, further suggesting that the Kevlar fibers effectively form fiber-bridging structures at the interface, which enhances the interfacial toughness and strengthens the adhesion between the matrix and fibers (Figure 10).

**Figure 11:**
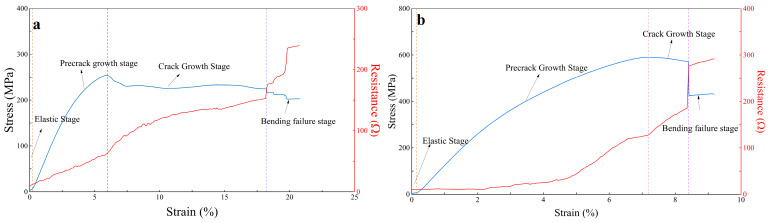
(**a**) Stress/resistance–strain curves of aramid fiber laminate with only LIG-coated; (**b**) Stress/resistance–strain curves of aramid fiber laminates containing LIG-coated and short Kevlar fibers.

The LIG-coated aramid fiber laminates serve two key purposes: Firstly, the laser treatment enhances the surface roughness of the aramid fibers, improving the adhesion between the panel and the epoxy resin, thereby increasing interlaminar fracture toughness. Secondly, the inherent piezoresistive properties of graphene enable the monitoring of delamination within the laminate interface. The variations in stress and resistance with strain for aramid fiber laminates containing LIG-coated are illustrated in Figure 11. From the figure, it is evident that there is a correlation between changes in stress and resistance as strain increases. Initially, the relationship between stress and strain is linear, followed by a transition to a nonlinear relationship. Simultaneously, resistance exhibits both linear and nonlinear growth patterns. During the linear phase, the integrity of the laminate structure is maintained, and the conductive pathways within the laminate remain intact, resulting in relatively stable resistance. As the system enters the nonlinear phase, pre-cracks start to propagate, leading to partial disintegration of graphene and affecting the conductive pathways, which accelerates the rate of resistance increase. This indicates a correlation between structural delamination and the rate of resistance change. When the stress reaches a critical value, cracks begin to propagate rapidly, resulting in the failure of the connections within the LIG layer, causing a significant increase in resistance and demonstrating a correlation between crack propagation and resistance change rate. Subsequently, the load experiences a sharp decline, indicating rapid expansion of interlaminar cracks. Concurrently, resistance rises sharply; when the stress stabilizes, the rate of resistance increase correspondingly decreases. Ultimately, bending failure of the laminate leads to a drop in stress, which corresponds to an increased rate of resistance change. In summary, LIG not only effectively monitors the initiation of delamination in laminates but also serves as a reliable indicator for crack propagation and bending failure.

### 3.3. In-Plane Mechanical Properties

The previous studies have confirmed that LIG and short Kevlar fibers significantly enhance the fracture toughness of aramid fiber-reinforced composite laminates. However, many methods for improving interlaminar toughness often come at the expense of in-plane performance [38,39,40]. Therefore, it is crucial to investigate the impact of LIG and short Kevlar fibers on the in-plane mechanical properties of the composites. This paper examines the effects of LIG and short Kevlar fibers on the in-plane mechanical properties of aramid fiber laminates through tensile testing.

**Figure 12:**
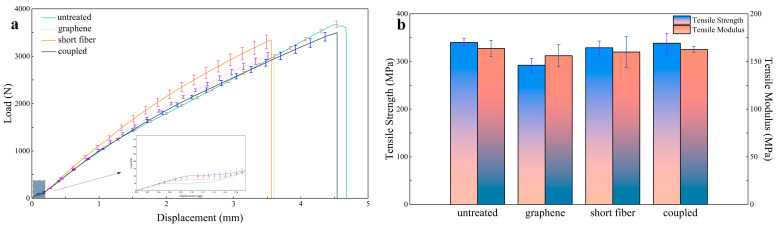
(**a**) Average load displacement curves of the four types of specimens; (**b**) Tensile strength and tensile modulus of the four types of specimens.

The average load displacement curves for the four types of specimens under tensile testing are shown in Figure 12. It can be observed that all four types of specimens undergo an elastic deformation phase followed by a plastic deformation phase. During the elastic deformation phase, the load displacement relationship increases linearly. Subsequently, in the plastic deformation phase, as the applied displacement load increases, the load exhibits a nonlinear growth trend, and fiber damage begins to occur. After reaching the maximum load that the specimen can withstand, the load starts to decrease, indicating that the specimen has reached its damage limit and ultimately fractures.

The magnified view of the average load displacement curve indicates that the laminated composites containing only LIG-coated aramid fiber and those with a combination of LIG-coated and short Kevlar fibers exhibit a prolonged linear growth phase. This suggests that, compared to the other two types of specimens, they are capable of maintaining a longer elastic phase. This is attributed to the fact that graphene, as a surface coating on aramid fibers, can effectively distribute stress over the fiber surfaces during tensile loading, reducing localized stress concentrations and ensuring that the entire laminate can uniformly withstand external forces during stretching. This capability allows it to endure greater loads without undergoing plastic deformation, thereby extending the elastic deformation phase. Furthermore, the LIG coating can partially constrain the sliding of aramid fibers, enhancing the overall structural integrity of the material at the microscale, resulting in improved linear elastic characteristics during tensile testing. Furthermore, the slope of the linear phase in the load displacement curve represents the tensile modulus of the laminate, with values of 163.65 MPa, 156.01 MPa, 159.94 MPa, and 162.48 MPa, all exhibiting variations within 5% (Figure 12b). This indicates that the incorporation of short Kevlar fibers and LIG did not significantly alter the tensile modulus of the laminate, resulting in comparable slopes during the linear phase of the curve.

An analysis of Table 3 reveals that the ultimate load for the untreated aramid fiber laminate, the laminate containing only LIG-coated, the laminate with only short Kevlar fibers, and the laminate combining both are 3745.63 N, 3220.62 N, 3624.86 N, and 3729.12 N, respectively. It is evident that the ultimate tensile load of the laminate containing only LIG-coated aramid fibers is significantly lower than that of the other three types. This indirectly indicates that the surface modification of aramid fibers using a 12 W laser has caused damage to the in-plane fibers, resulting in the formation of surface defects that compromise the tensile properties of the fibers. Conversely, incorporating short Kevlar fibers into the laminate containing LIG-coated aramid fibers can partially compensate for the reduction in fiber quantity due to laser treatment, ensuring that the overall weight of the fiber fabric does not experience significant loss. Therefore, the laminate that combines LIG-coated with short Kevlar fibers can maintain the in-plane mechanical properties of the structure effectively.

Figure 12b illustrates the tensile strengths of the four types of specimens. The tensile strength of the untreated aramid fiber laminate (339.74 MPa) is comparable to that of the short Kevlar fiber laminate (328.79 MPa) and the laminate combining LIG-coated with short Kevlar fibers (338.24 MPa), with variations within 5%. This indicates that both the laminate containing only short Kevlar fibers and the laminate with LIG-coated and short Kevlar fibers have a limited impact on the tensile properties of the aramid fiber laminate. This limitation is attributed to the relatively low number of short fiber interlayers, which do not significantly affect the thickness of the laminate, thereby resulting in no change in the in-plane mechanical properties of the composite material. Conversely, the tensile strength of the laminate containing only LIG is 292.12 MPa, representing a reduction of 14.02% compared to the untreated laminate. This decrease is attributed to the transformation of aramid fiber surfaces into LIG and gaseous substances upon laser irradiation, leading to a reduction in the number of aramid fibers in both the longitudinal and transverse directions, which in turn decreases the overall density of the aramid fibers and their load-bearing capacity. Therefore, although LIG can significantly improve the fracture toughness of the laminate, this comes at the expense of in-plane mechanical strength. However, the incorporation of short Kevlar fibers effectively mitigates this loss of strength, preserving the in-plane mechanical performance of the composite material.

**Figure 13:**
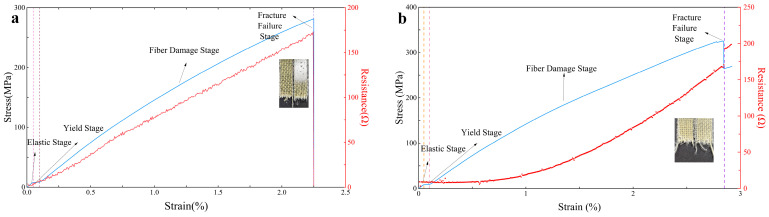
(**a**) Stress/resistance–strain curves of the aramid fiber laminate with only LIG-coated; (**b**) Stress/resistance–strain curves of the aramid fiber laminate containing LIG-coated and short Kevlar fibers.

To monitor the damage evolution of the specimens in real time during the tensile testing, load displacement and load were recorded using a universal testing machine for the laminate containing LIG. Simultaneously, the four-probe method was employed to measure the resistance in situ (Figure 13). In the initial stage of displacement loading, the specimens were in the elastic deformation stage, and the stress–strain curve demonstrated a linear relationship; correspondingly, the resistance increased approximately linearly. This is attributed to the laser treatment that transforms the outer fibers of the aramid fibers’ surface into piezoresistive graphene. During the application of tensile force, the number of carbon–carbon contacts on the surface of the aramid fibers decreases, resulting in an increase in resistance. At this stage, the laminate experiences minimal damage, maintaining an intact conductive pathway and relatively stable resistance values. As displacement increases, the specimens transition into the plastic deformation phase, where changes in resistance align with stress variations, both exhibiting a nonlinear growth trend. This behavior is due to the emergence of micro-damage, such as cracks in the laminate, which leads to localized breakage of the graphene pathways, causing the rate of resistance increase to accelerate. In this phase, the deformation of the specimens becomes irreversible, and the separation between graphene surfaces is also irreversible. In the later stages of loading, the specimens reach critical load, leading to progressive failure of the fiber reinforcement. The damage intensifies, culminating in macroscopic failure of the laminate, where the conductive pathways on each layer’s surface separate, resulting in an increased rate of resistance change and a significant spike in resistance values (Figure 13b). For another specimen, a sudden drop in resistance was observed due to wire fracture caused by the failure during displacement loading (Figure 13a).

## 4. Conclusions

This study investigates the application of LIG coating on the surface of aramid fibers, using short Kevlar fibers as interlayer toughening agents in aramid fiber/epoxy resin composites. The effects of LIG and short Kevlar fibers on the interfacial toughness and damage detection of aramid fiber/epoxy resin composites were systematically explored. The following conclusions were drawn:

(1) Both LIG and short Kevlar fibers significantly improve the interfacial toughness of aramid fiber-reinforced composite laminates. The synergistic effect results in a Mode II fracture toughness of 9460.79 kJ/m^2^, representing a substantial 381.60% increase relative to untreated composites. In comparison, LIG alone enhances the toughness by 60.48%, while short Kevlar fibers contribute a 43.00% improvement. When compared to the existing literature on carbon fiber/epoxy composites, the toughening effects of LIG (69% enhancement) and short Kevlar fibers (61.8% enhancement) are notably lower than the 381.60% increase observed in aramid fiber composites. Therefore, the incorporation of LIG and short Kevlar fibers constitutes an effective approach for improving the interfacial toughness of aramid fiber-reinforced composites, significantly enhancing the material’s interfacial toughness and structural integrity.
(2) Short Kevlar fibers can compensate for the reduction in in-plane mechanical properties caused by the LIG-coating. The tensile strength of composites containing only the LIG-coated is 292.12 MPa, which represents a decrease of 14.02% compared to the untreated composites. In contrast, the tensile strength of the combined system is 338.24 MPa, with a variation maintained within 5%. These results indicate that the introduction of short Kevlar fibers mitigates the decline in tensile load-bearing capacity resulting from the laser treatment of aramid fiber surfaces, thereby preserving the in-plane mechanical performance of the structure.
(3) The toughening mechanism of aramid fiber-reinforced composites containing LIG and short Kevlar fibers was investigated using SEM. The LIG-coating enhances toughness by increasing the surface roughness of the fibers and promoting fiber-matrix interlocking, while short Kevlar fibers improve toughening effects by introducing complex crack paths and energy absorption mechanisms through fiber pull-out, fiber fracture, and fiber bridging. The combination of both components further enhances the fracture toughness of the laminate by improving interfacial bonding and crack propagation pathways.
(4) LIG can effectively monitor the delamination and tensile damage processes of laminates. Through resistance changes, it allows for real-time tracking of interlayer damage and tensile development. During the Mode II tests, the resistance exhibited both linear and nonlinear relationships with strain, showing a significant increase in the rate of resistance change at the onset of delamination and during crack propagation. This indicates a correlation between resistance changes and the occurrence of delamination and crack growth. During the tensile tests, resistance initially increased linearly in the elastic phase and then nonlinearly in the plastic phase. A sudden increase in the rate of resistance change was observed when the specimen exhibited tensile fracture damage, suggesting a relationship between resistance changes and the emergence of damage. Thus, LIG provides a novel method for in situ damage detection within structures.

## Figures and Tables

**Table 1:** Ultimate load and fracture toughness of four types of specimens.

Type	Ultimate Load (N)	Fracture Toughness (KJ × m^−2^)
Untreated	752.60 ± 32.45	1964.46 ± 35.11
LIG	947.42 ± 33.92 (25.89%)	3152.53 ± 266.43 (60.48%)
Short Kevlar fibers	1333.47 ± 233.10 (752.60%)	2809.48 ± 647.82 (43.00%)
LIG–short Kevlar fibers	1757.91 ± 513.91 (133.58%)	9460.49 ± 1704.91 (381.79%)

**Table 2:** Comparison of fracture toughness change rates from different studies.

Type	Fracture Toughness Change Rate
LIG [23]	69%
Short Kevlar fibers [37]	61.8%
LIG–short Kevlar fibers	381.79%

**Table 3:** Ultimate load, tensile strength, and tensile modulus of four types of specimens.

Type	Ultimate Load (N)	Tensile Strength (MPa)	Tensile Modulus (MPa)
Untreated	3745.63 ± 98.45	339.74 ± 8.93	163.65 ± 8.46
LIG	3220.62 ± 160.33	292.12 ± 14.54 (−14.02%)	156.01 ± 11.64 (−4.67%)
Short Kevlar fibers	3624.86 ± 156.13	328.79 ± 14.16 (−3.22%)	159.93 ± 16.29 (−2.27%)
LIG–short Kevlar fibers	3729.12 ± 224.55	338.24 ± 20.37 (−0.44%)	162.48 ± 3.28 (−0.71%)

## Data Availability

The raw data supporting the conclusions of this article will be made available by the authors on request.
